# Efficacy and Safety of Immune Checkpoint Inhibitor Rechallenge in the Treatment of Esophageal Squamous Cell Cancer

**DOI:** 10.7150/jca.104380

**Published:** 2025-01-01

**Authors:** Xiaojing Zhang, Jingze Zhang, Junyi He, Xiao Zhong, Jinming Yu, Linlin Wang

**Affiliations:** 1 Department of Radiation Oncology, Shandong Cancer Hospital and Institute, Shandong First Medical University and Shandong Academy of Medical Sciences, Jinan, Shandong, China.; 2 Shandong University Cancer Center, Cheeloo College of Medicine, Shandong University, Jinan, Shandong, China.

**Keywords:** esophageal squamous cell carcinoma, immune checkpoint inhibitor, Immunotherapy, rechallenge, retrospective analysis

## Abstract

**Background:** Rechallenge with immune checkpoint inhibitors (ICI) shows promise in various cancers, but data in esophageal squamous cell carcinoma (ESCC) is limited. This study aimed to evaluate the efficiency and safety of ICI rechallenge in ESCC.

**Materials and Methods:** This multicenter study analyzed ESCC patients rechallenged with ICI from January 2020 to March 2023 across two medical institutions. Patients were divided into rechallenge (R) and non-rechallenge (NR) groups. Key outcomes studied were progression-free survival (PFS), overall survival (OS), and safety.

**Results:** Among 329 included ESCC patients, 211 were in the R group and 118 in the NR group, with a median follow-up of 17.1 months. The R group exhibited significantly prolonged median PFS (4.7 vs. 3.2 months; p <.001) and OS (9.3 vs. 6.2 months; p <.001) compared to the NR group. Notably, for patients who initially received radiotherapy, the R group showed significantly longer mPFS (5.1 vs. 3.2 months; p <.001) and mOS (10.4 vs. 5.9 months; p <.001). Incidences of all-grade (64.5% vs. 66.1%; p = .764) and grade ≥3 adverse events (17.5% vs. 18.6%; p = .802) did not significantly differ between groups.

**Conclusion:** ICI rechallenge demonstrates efficacy and manageable safety in ESCC, particularly post-radiotherapy.

## Introduction

Global cancer statistics from 2020 indicate that esophageal cancer accounts for the sixth highest number of cancer-induced deaths 1. East Asia, especially China, has the highest incidence of esophageal cancer 2. Esophageal squamous cell carcinoma (ESCC) constitutes roughly 90% of all esophageal cancers 3. Most patients with esophageal cancer are diagnosed with advanced disease, and the 5-year survival rate is 26% 4.

Recently, immune checkpoint inhibitors (ICIs) remarkably prolonged OS in patients with malignancies, including those with esophageal cancer 5-8. Based on the findings from to the ESCORT, KEYNOTE-181, and ATTRACTION-3 studies 7,9,10, programmed death receptor 1 (PD-1) inhibitor monotherapy has shown better anti-tumor efficacy to chemotherapy in the second-line treatment of advanced esophageal cancer, with a favorable safety profile. Trials like ESCORT-1st, KEYNOTE-590, and CheckMate648 have propelled the search for first-line immunotherapy in advanced cases and shown significant efficacy. According to the findings, the combination of ICIs with chemotherapy has been advised as the preferred first-line treatment for advanced esophageal cancer, rather than chemotherapy alone 5-8.

However, only a portion of patients experience a durable response to first-line immunotherapy, and resistance to therapy and disease progression can still occur as time passes. When these occur, there are no established strategies to overcome drug resistance 11. Clinical researches show that repeated use of immunotherapy may be a more favorable option than traditional chemotherapy and radiotherapy 12. Some patients might benefit from an immunotherapy rechallenge. Successful ICI rechallenge has been reported in patients with several solid tumors, such as melanoma 13-16, lung cancer 17, hepatocellular 18 and renal cell carcinoma 19,20. However, as far as we know, there is currently a lack of data to approach this strategy in patients with ESCC. Our study aimed to evaluate the efficacy and safety of ICI rechallenge in patients with ESCC who discontinued first-line ICI treatment.

## Materials and methods

### Data collection

This study adhered to the principles outlined in the Declaration of Helsinki (revised in 2013). Due to the retrospective nature of the study, informed consent was deemed unnecessary. Data from patients with ESCC receiving PD-1/PD-L1 inhibitors as first-line treatment at Shandong Cancer Hospital and Institute and Shandong Provincial Hospital from January 2020 to April 2023 were retrospectively collected. Inclusion criteria were: (i) histologically confirmed ESCC; (ii) diagnosis of recurrent or metastatic ESCC; (iii) first-line treatment with anti-PD-1/PD-L1 inhibitors; (iv) discontinuation of anti-PD-1/PD-L1 inhibitors for any reason (disease progression, development of adverse events [AEs], protocol completion); (v) first-line treatment including at least two cycles of PD-1/PD-L1 inhibitors. The exclusion criteria were: (i) no tumor evaluation performed and (ii) presence of other primary tumor types. The enrolled patients were categorized into two groups: (i) the rechallenge group (R group, n=211), including patients who discontinued anti-PD-1/PD-L1 inhibitors and subsequently received anti-PD-1/PD-L1 inhibitors again and (ii) the non-rechallenge group (NR group, n=118), including patients who were not retreated with anti-PD-1/PD-L1 inhibitors. Patient data included age, sex, smoking and alcohol consumption history, Eastern Cooperative Oncology Group performance status (ECOG PS) score, lung/liver/bone metastases, best response to initial immunotherapy, reason for discontinuation of ICI-1, time to relapse after ICI-1, history of radiotherapy, first- and second-line therapeutic schedule, clinical T-stage and N-stage according to the AJCC TNM staging system (eighth edition).

### Efficacy evaluation

The primary endpoints were PFS, OS and safety. PFS was defined as the duration time from the start of the second-line ICI treatment or another therapy to tumor progression or death from any causes. OS referred to the period from the start of the second-line treatment to death. The secondary endpoints were objective response rate (ORR) and disease control rate (DCR). Assessments of tumor response, including complete response (CR), partial response (PR), stable disease (SD), and progressive disease (PD), were conducted in accordance with the RECIST 1.1 criteria 21. ORR was determined by the proportion of individuals who obtained a CR and PR. DCR was determined by the proportion of individuals who obtained a CR, PR, or SD. The last follow-up date was November 30, 2023.

### Statistical analysis

Baseline characteristics were described using the median and interquartile range (IQR) for continuous variables and frequency and percentage for categorical variables. Qualitative variables were analyzed using the chi-squared test or Fisher exact test. PFS and OS were assessed using the Kaplan-Meier method, and differences were compared using the log-rank test. Univariate and multivariate Cox regression analyses were conducted to identify predictors of PFS and OS, with hazard ratios (HR) and 95% confidence intervals (95% CIs) reported. Variables that reached a significance threshold of P<.05 in the univariate assessment were then included in the multivariate Cox regression model. Statistical significance was set at a two-tailed P-value below 0.05. The analyses were conducted utilizing the R statistical software, version 4.3.1.

## Results

### Patient clinical characteristics

Totally 329 patients with ESCC treated with ICIs met the criteria for inclusion. Among these, 211 and 118 were included in the R and NR groups, respectively. The baseline clinical features of both groups were well-matched (Table 1). There were 298 male patients (90.6%) and the median age of the overall study patients was 62 years. 172 (52.3%) patients with a smoking history. Most patients (96.4%) had an ECOG PS score of 0-1. Most patients received ICI combination therapy before first progression (95.7%), and 4.3% received immune monotherapy. Fewer patients in the NR group (71.6%) had received prior radiotherapy than those in the R group (77.1%); however, the difference did not reach statistical significance.

### Efficacy evaluation

**Figure 1:**
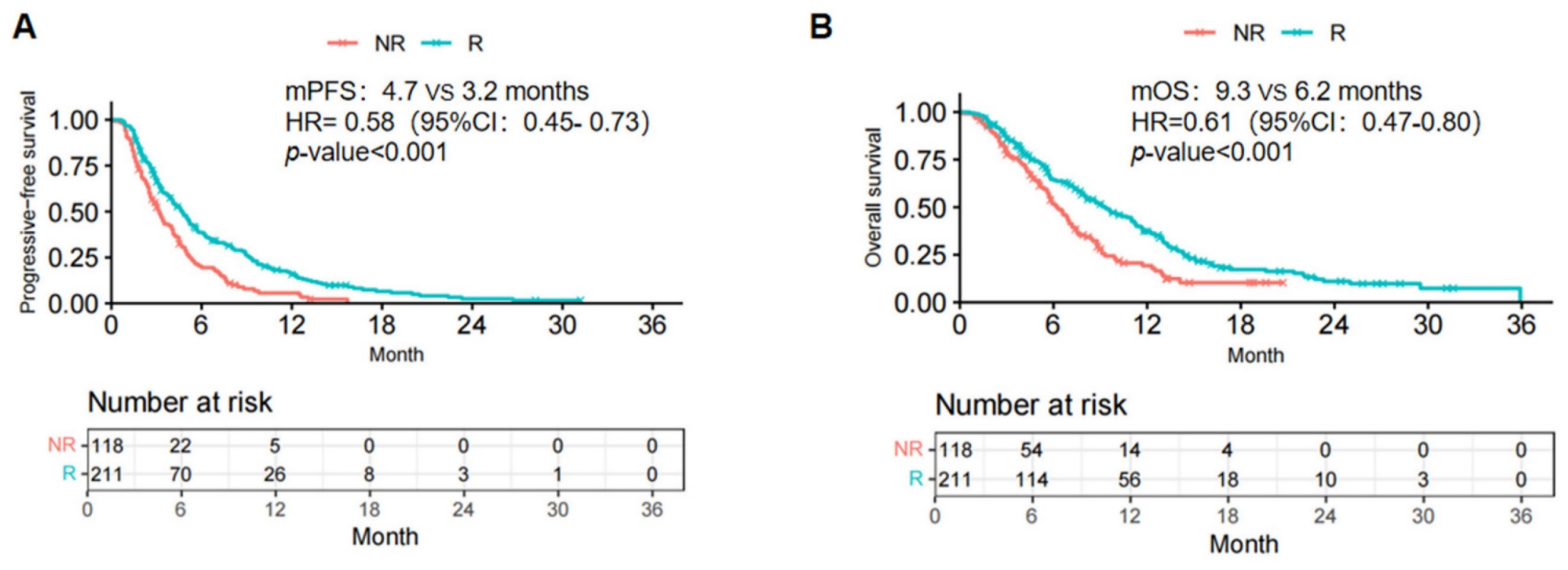
Kaplan-Meier curves of PFS (A) and OS (B) of ICI rechallenge in the total study population. R, rechallenge group; NR, non rechallenge group; mPFS, median progression-free survival; mOS, median overall survival; HR, hazard ratio; CI, confidence interval.

The median follow-up duration was 17.1 months. As of the follow-up date, 290 patients (88.1%) had experienced disease progression, including 180 (85.31%) and 110 (93.22%) in the R and NR groups, respectively. Additionally, 239 patients (72.64%) died (148 [70.14%], R group; 91 (77.12%), NR group). The mPFS and mOS were statistically prolonged in patients in the R group compared to those in the NR group within the overall study cohort (mPFS: 4.7 vs. 3.2 months; HR =0.58; 95% confidence interval [CI]: 0.45-0.73; *P* <.001; Fig. 1A; mOS: 9.3 vs. 6.2 months; HR =0.61; 95% CI: 0.47-0.80; *P* <.001; Fig. 1B).

In the R group, no patients experienced CR (0%); 57, PR (23.7%); 107, SD (50.7%); and 54, PD (25.6%) (Table 2). Compared to the NR group, the R group exhibited significantly elevated ORR (23.7% vs. 9.3%; P=.001) and DCR (74.4% vs. 55.1%; *P* <.001).

The multivariate analysis indicated that anti-PD-1/PD-L1 inhibitor rechallenge (HR =0.55; 95% CI: 0.43-0.70, p<.001); ECOG PS score≥2 (HR =2.56; 95% CI: 1.39-4.72, P=.003), discontinuation due to disease progression (HR =1.72; 95% CI: 1.15-2.57; P=.008), and N3 stage (HR =1.68; 95% CI: 1.29-2.19; P<.001) were associated with PFS (Table 3). In addition, anti-PD-1/PD-L1 inhibitor rechallenge (HR =0.58; 95% CI: [0.44-0.76]; *P* <.001), ECOG PS score≥2 (HR =1.95; 95% CI: 1.06-3.62; P=.032), discontinuation due to disease progression (HR =1.84; 95% CI: 1.16-2.93; P=.010), and N3 stage (HR =1.73; 95% CI: 1.31-2.30; P<.001) were associated with OS (Table 4).

### Subgroup analysis

**Figure 2:**
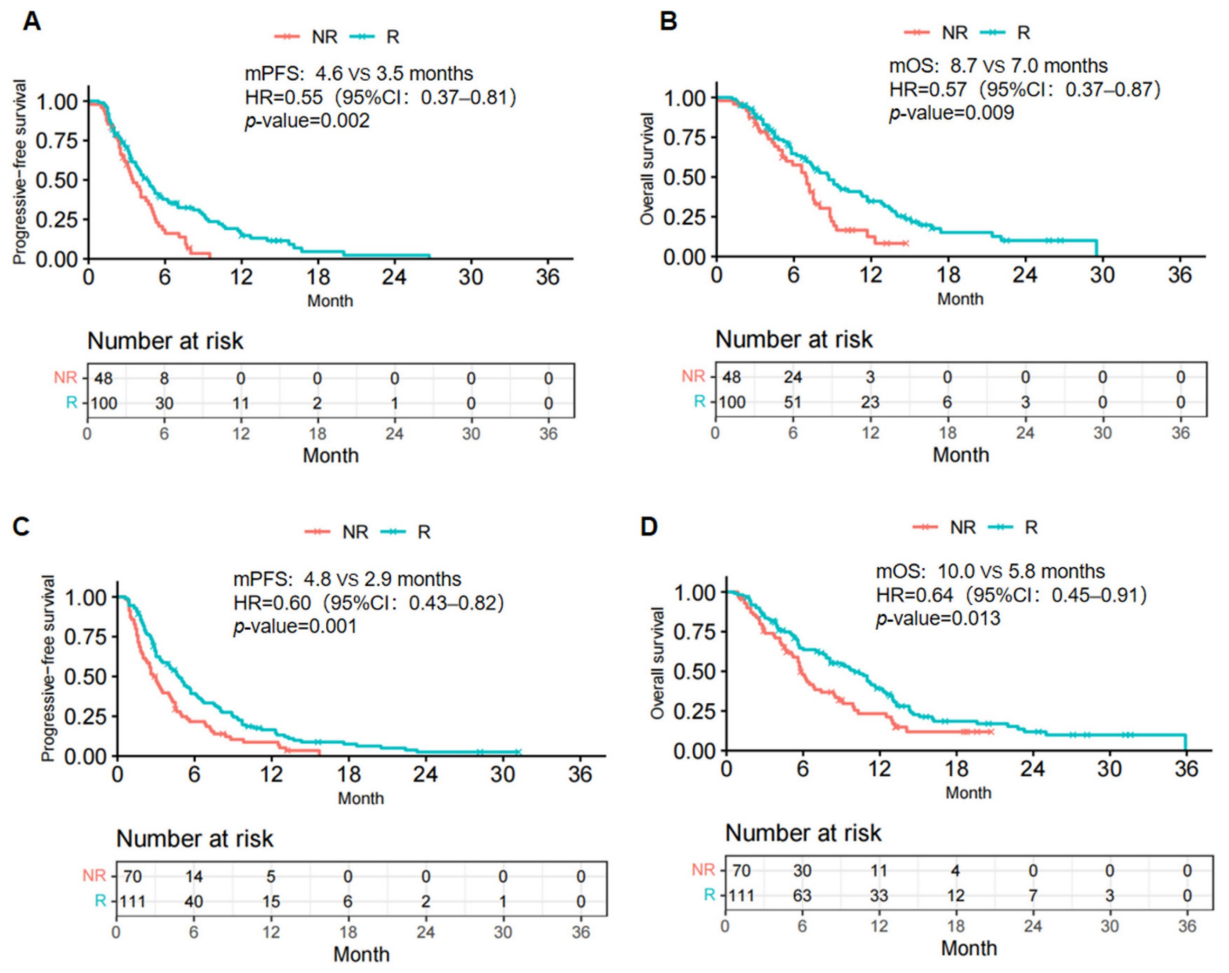
Kaplan-Meier curves of PFS (A, C) and OS (B, D) from data in the CR/PR response (A, B) and SD/PD response (C, D) to initial immunotherapy subgroups. R, rechallenge group; NR, non-rechallenge group; mPFS, median progression-free survival; mOS, median overall survival; HR, hazard ratio; CI, confidence interval.

In the subgroup analysis, in those achieving CR/PR response to initial immunotherapy, ICI rechallenge exhibited significant advantages in both PFS and OS (mPFS: 4.6 vs. 3.5 months, HR=0.55, 95% CI: 0.37-0.81, p = .002; mOS: 8.7 vs. 7.0 months, HR=0.57, 95% CI: 0.37-0.87, p = .009) (Fig. 2A, B). Interestingly, in the subgroup achieving SD/PD response, the survival analysis demonstrated similar results (mPFS: 4.8 vs. 2.9 months, HR: 0.60, 95% CI: 0.43-0.82, p = .001; mOS: 10.0 vs. 5.8 months, HR: 0.64, 95% CI: 0.45-0.91, p = .013) (Fig. 2A, B). Therefore. ICI rechallenge resulted in clinical benefits regardless of the initial treatment response in this study cohort.

**Figure 3:**
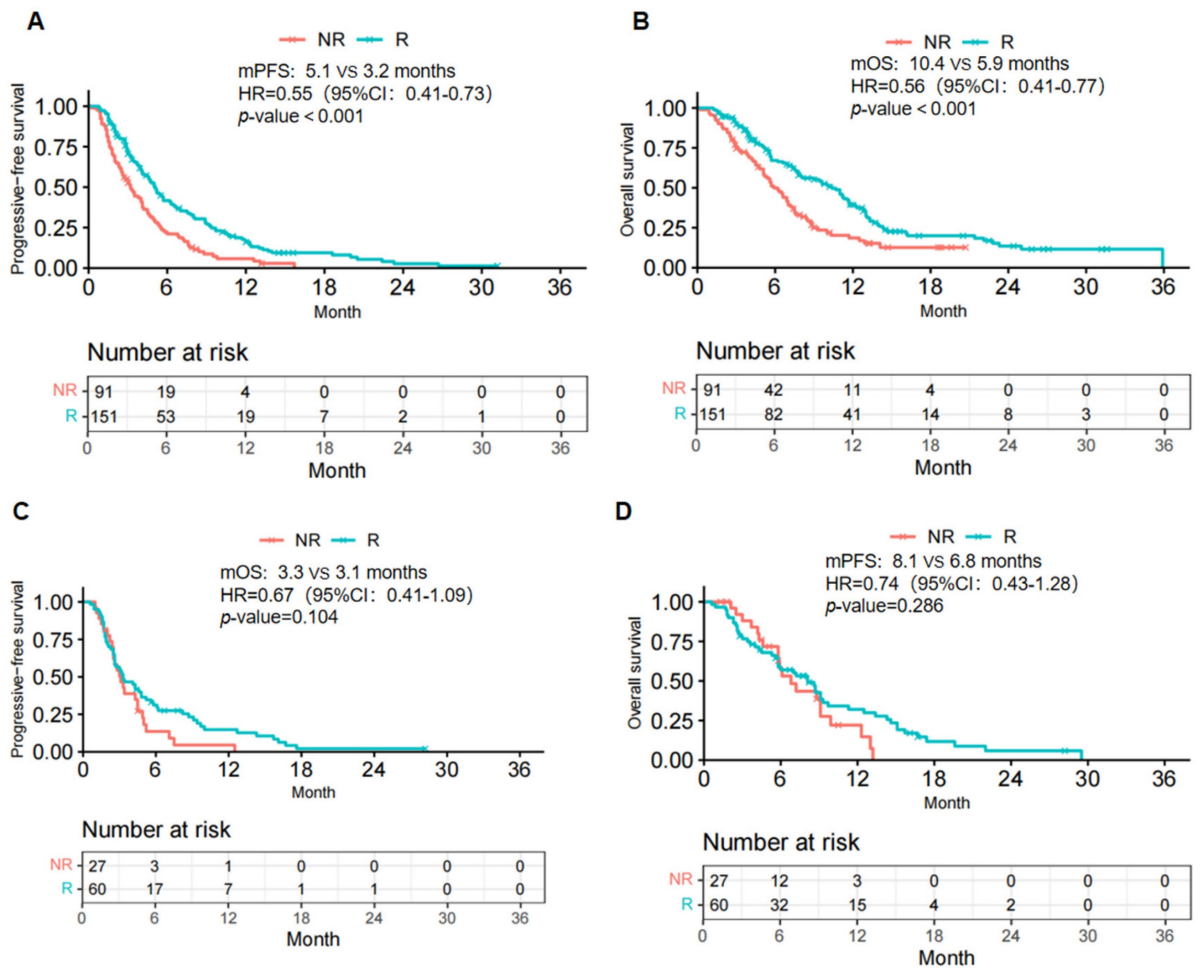
Kaplan-Meier curves of PFS (A, C) and OS (B, D) from data in the previously radiotherapy (A, B) and non-radiotherapy (C, D) subgroups. R, rechallenge group; NR, non-rechallenge group; mPFS, median progression-free survival; mOS, median overall survival; HR, hazard ratio; CI, confidence interval.

Subgroup analyses were conducted in the R and NR groups to investigate the effect of previous radiotherapy on ICI rechallenge. For patients who accepted radiotherapy in the initial treatment, the R group demonstrated a notable increase in mPFS (5.1 vs. 3.2 months; HR=0.55; 95% CI: 0.41-0.73, p <.001) and mOS (10.4 vs. 5.9 months; HR=0.56; 95%CI: 0.41-0.77, p <.001) (Fig. 3A, B). For patients who did not receive radiotherapy in the initial treatment, although the mPFS and mOS in the R group were longer than those in the NR group (mPFS: 3.3 vs. 3.1 months, HR: 0.67, 95% CI: 0.41-1.09, p = .104; mOS: 8.1 vs. 6.8 months, HR: 0.74, 95%CI: 0.43-1.28, p = .286), these differences did not show statistical significance (Fig. 3C, D).

### Safety of rechallenge with ICIs

Treatment-related adverse events (TRAEs) were observed in 136 out of 211 patients (64.5%) in the R group and 78 out of 118 patients (66.1%) in the NR group (p=.764). The most common AEs were diarrhea (n = 41[19.4%]), vomiting (n = 39[18.5%]), fatigue (n = 38[18.0%]), and neutropenia (n = 36[17.1%]) in the R group and fatigue (n = 21[17.8%]) and neutropenia (n = 20[16.9%]) in the NR group. Grade 3 or higher TRAEs occurred in 37 patients (17.5%) within the R group and 22 patients (18.6%) within the NR group (p=.802) (Table 5). Fifteen (7.1%) and eight patients (6.8%) discontinued at least one treatment component because of TRAEs (Supplementary Table 1). No treatment-related deaths occurred. Among the six patients who discontinued ICI therapy due to AEs, four accepted ICI rechallenge, one of whom experienced grade 3 diarrhea, which was also observed during first-line immunotherapy.

## Discussion

We performed this multicenter retrospective cohort study in patients with recurrent and metastatic ESCC and demonstrated the efficacy of ICI rechallenge after first-line immunotherapy. Among patients who discontinued the first-line treatment regimen including anti-PD-1/L1 antibody, rechallenge of ICI treatment achieved a higher response rate (ORR: 23.7% vs. 9.3%; P=.001; DCR: 74.4% vs. 55.1%; P<.001) and longer survival (mPFS: 4.7 vs. 3.2 months, HR =0.58, 95% CI: 0.45- 0.73, P<.001; mOS: 9.3 vs. 6.2 months, HR =0.61, 95% CI: 0.47-0.80, P<.001).

Based on the results of the subgroup analysis conducted according to the best response to the first-line immunotherapy, regardless of the initial response to first-line treatment, significant statistical differences still existed in the PFS and OS between the rechallenge and non-rechallenge groups. In terms of chemotherapy and targeted therapy, resistance is defined as disease progression after a duration of relevant medicine use that may not be applicable to immune treatment. Several studies on other solid tumors, such as lung cancer and renal cell cancer, have shown that the efficacy of ICI rechallenge is independent of the response to the first course of ICI 22-25. This may be due to a delay in the immune response caused by the time required for the immune system to initiate an anti-tumor response 26,27. Therefore, patients with ESCC who fail to achieve satisfactory tumor regression with initial immunotherapy may still benefit from the rechallenge treatment.

The subgroup analysis revealed that radiotherapy as the initial treatment is a critical factor in enhancing the efficacy of ICI rechallenge. In patients who initially underwent radiotherapy, the reintroduction of immunotherapy resulted in a significant survival benefit. However, this benefit was unclear in patients who did not receive radiotherapy. This finding is consistent with the commonly recognized synergistic effects of immunotherapy and radiotherapy. Besides its direct tumoricidal effects, ionizing radiation is deeply involved in the anti-tumor immune response. Relevant mechanisms include the release of tumor-associated antigens 28, activation of dendritic cells 29, upregulation of cytokines and chemokines 30, normalization of the tumor vasculature 31. Preclinical studies have shown that radiotherapy-induced remodeling of the tumor immune microenvironment helps build immune memory and overcome immune evasion, enhancing the anti-tumor efficiency of subsequent immunotherapy 32,33. The enhancement of immunotherapy provided by prior irradiation was also corroborated by reliable clinical evidence, namely, the Pacific trial, which established the value of ICI after chemoradiotherapy in local advanced Non-small cell lung cancer (NSCLC) 34. Similar conclusions have been drawn by Garon *et al.* 35. Accordingly, we inferred that initial radiotherapy is a favorable factor for immunotherapy rechallenge.

The findings of the multivariate analysis revealed that patients with better ECOG PS scores and earlier N stages benefited significantly from ICI rechallenge. Physical function and immune status are still crucial factors for the rechallenge of immunotherapy, which is consistent with previous findings. Imbalance of circulating T-lymphocyte subpopulations correlates with ECOG PS scores in patients with gastric cancer 36. A low ECOG-PS score serves as a negative prognostic indicator concerning the clinical outcomes of initial ICI therapy in NSCLC patients 37-40. Along with the disease extent, the N stage also reflects the extent of impairment in the efficiency of the anti-tumor immune response. A solid and convincing study found that dynamic CD8+ T-cell responses in normal lymph nodes, which are critical to immunotherapy, are disrupted in metastatic lymph nodes 41. A retrospective clinical analysis also found that retaining more normal lymph nodes after surgery was associated with elevated immunotherapy efficacy 42. The expression status of PD-L1 is considered one of the most reliable biomarkers for ICI treatments. Unfortunately, owing to the unavailability of PD-L1 status, we did not conduct a relevant analysis.

Our results showed similar rates of all-grade and high-grade TRAEs in both the groups. However, the types of AEs varied between the two groups, with gastrointestinal reactions and hematologic toxicity predominating in the R group and hematologic toxicity predominating in the NR group. Of the four patients who underwent ICI rechallenge after discontinuation due to AEs, one patient experienced a grade 3 gastrointestinal AE, similar to his initial AE. For patients who received radiotherapy during initial treatment, the incidence of AEs was similar to that of patients who did not receive radiotherapy, and no new AEs were identified. The Keynote001 study also demonstrated that there were no statistical differences in the frequency of pulmonary toxicity between patients who did and did not previously receive thoracic radiotherapy 35. Our study shows that ICI rechallenge has acceptable safety profiles regardless of whether patients received radiotherapy during their initial treatment. Close monitoring and adherence to standard treatment protocols are essential for identifying and managing the toxic effects associated with ICI rechallenge.

Our findings further contribute additional clinical evidence on the efficacy and safety of ICI rechallenge in patients with recurrent and metastatic ESCC. However, our study still has limitations. First, its retrospective design may have introduced recall bias and data gaps. Second, the lack of standardized rechallenge combination regimens may have affected the results and safety profiles. ICIs have only been approved as first-line immunotherapy options for esophageal cancer in China for a limited period. We have collected all currently eligible cases and will continue to expand the number of cases. In addition, prospective studies (NCT03736863) are ongoing, and further prospective clinical trials is warranted to explore the efficacy and safety of rechallenging with anti-PD-1/PD-L1 inhibitors.

In conclusion, rechallenge with ICIs is a viable option for patients with ESCC considering its encouraging efficacy and manageable safety, particularly in patients previously treated with radiotherapy. Further prospective trails are required to confirm these results.

## Supplementary Material

Supplementary table.

## Figures and Tables

**Table 1:** Baseline characteristics of the patients.

	All patients (n = 329)	Rechallenge(n=211)	Non-rechallenge(n=118)	p-value
Age				
Median age (range), years	62(56-68)	62(57-69)	62(55-67)	0.116
≤60	133(40.4%)	92(43.6%)	41(34.7%)	
>60	196(59.6%)	119(56.4%)	77(65.3%)	
Sex				
Male	298(90.6%)	191(90.5%)	107(90.7%)	0.963
Female	31(9.4%)	20(9.5%)	11(9.3%)	
Smoking history				
Ever	172(52.3%)	106(50.2%)	66(55.9%)	0.321
Never	157(47.7%)	105(49.8%)	52(44.1%)	
Drinking history				
Ever	172(52.3%)	106(50.2%)	66(55.9%)	0.321
Never	157(47.7%)	105(49.8%)	52(44.1%)	
Best response to first line				
PR	148(45.0%)	100(47.4%)	48(40.7%)	0.362
SD	123(37.4%)	73(34.6%)	50(42.4%)	
PD	58(17.6%)	38(18.0%)	20(16.9%)	
Liver metastasis				
Yes	52(15.8%)	34(16.1%)	18(15.3%)	0.838
No	277(84.2%)	177(83.9%)	100(84.7%)	
Lung metastasis				
Yes	36(10.9%)	22(10.4%)	14(11.9%)	0.689
No	293(89.1%)	189(89.6%)	104(88.1%)	
Bone metastasis				
Yes	19(5.8%)	13(6.2%)	6(5.1%)	0.688
No	310(94.2%)	198(93.8%)	112(94.9%)	
ECOG PS				
0-1	317(96.4%)	202(95.7%)	115(97.5%)	0.424
2	12(3.6%)	9(4.3%)	3(2.5%)	
Discontinuation reason				
Disease progression	296(90.0%)	191(90.5%)	105(89.0%)	0.893
Toxicity	6(1.8%)	4(1.9%)	2(1.7%)	
Others	27(8.2%)	16(7.6%)	11(9.3%)	
Time to relapse after ICI-1 (months)				
<3	202(61.40%)	130(61.61%)	72(61.02%)	0.915
≥3	127(38.60%)	81(38.39%)	46(38.98%)	
Radiotherapy				
Yes	242(73.6%)	151(71.6%)	91(77.1%)	0.273
No	87(26.4%)	60(28.4%)	27(22.9%)	
Treatment regimens of first-line				
ICIs monotherapy	14(4.3%)	7(3.3%)	7(5.9%)	0.442
ICIs with Chemotherapy	297(90.3%)	194(91.9%)	103(87.3%)	
ICIs with anti-VEGF	4(1.2%)	3(1.4%)	1(0.8%)	
ICIs combined with chemotherapy plus anti-angiogenesis therapy	14(4.3%)	7(3.3%)	7(5.9%)	
Treatment regimens of second-line				
ICIs monotherapy	17(5.17%)	17(8.06%)	0(0%)	<0.001
Chemotherapy with/without ICIs	232(70.52%)	137(64.93%)	95(80.51%)	
Anti-VEGF with/without ICIs	46(13.98%)	37(17.54%)	9(7.63%)	
Chemotherapy plus anti-angiogenesis therapy with/without ICIs	34(10.33%)	20(9.48%)	14(11.86%)	
Clinical T stage				
0-2	47(14.29%)	31(14.69%)	16(13.56%)	0.778
3-4	282(85.71%)	180(85.31%)	102(86.44%)	
Clinical N stage				
0-2	239(72.64%)	157(74.41%)	82(69.49%)	0.337
3	90(27.36%)	54(25.59%)	36(30.51%)	

Abbreviations: ICI: immune checkpoint inhibitor; PR: partial response; SD: steady disease; PD: progressive disease; ECOG PS: Eastern Cooperative Oncology Group Performance Status

**Table 2:** Cox proportional hazards and logistic regression models for progression-free survival (PFS).

Factors	PFS (Univariate analysis)				PFS (Multivariate analysis)	
		HR (95%CI)	p-value	HR (95%CI)		p-value
rechallenge		0.58(0.45- 0.73)	<0.001	0.55(0.43-0.70)		<0.001
Age (≥65)		0.94(0.73-1.20)	0.602			
Male gender		1.45(0.95- 2.2)	0.082			
ECOG PS≥2		2.23(1.2-4.10)	0.010	2.56(1.39-4.72)		0.003
Smoking history		1.14(0.91-1.45)	0.261			
Drinking history		1.14(0.90-1.44)	0.278			
Lung metastasis		0.91(0.63-1.31)	0.624			
Bone metastasis		1.26(0.77- 2.06)	0.357			
Liver metastasis		1.30(0.96-1.77)	0.095			
Best response to first line						
PR		1				
SD		1.010(0.78-1.31)	0.938			
PD		1.05(0.76-1.44)	0.773			
Primary tumor location						
Cervical		1				
Upper		1.05(0.54-2.04)	0.876			
Middle		1.08(0.58-2.02)	0.798			
Lower		0.95(0.51-1.77)	0.878			
Radiotherapy		0.85(0.66-1.10)	0.214			
Treatment regimens of first-line						
ICIs monotherapy		1				
ICIs with Chemotherapy		1.28(0.69-2.35)	0.435			
ICIs with anti-VEGF		1.81(0.50-6.56)	0.364			
ICIs with both Chemotherapy and anti-VEGF		1.40(0.60-3.24)	0.440			
Discontinuation due to disease progression		1.75(1.18-2.61)	0.006	1.72(1.15-2.57)		0.008
Distant organ metastasis		1.15(0.90-1.47)	0.260			
Time to relapse after ICI-1 (months)						
<3		1				
≥3		0.90(0.72-1.12)	0.327			
Clinical T stage						
0-2		1				
3-4		1.03(0.75-1.40)	0.862			
Clinical N stage						
0-2		1				
3		1.76(1.35-2.29)	<0.001	1.68(1.29-2.19)		<0.001
						

PR: partial response; SD: steady disease; PD: progressive disease; ECOG PS: Eastern Cooperative Oncology Group Performance Status; HR: hazard ratio; CI: confidence interval.

**Table 3:** Cox proportional hazards and logistic regression models for overall survival (OS)

Factors	OS (Univariate analysis)				OS (Multivariate analysis)	
		HR (95%CI)	p-value	HR (95%CI)		p-value
rechallenge		0.61(0.47-0.80)	<0.001	0.58(0.44-0.76)		<0.001
Age (≥65)		0.97(0.73-1.28)	0.822			
Male gender		1.46(0.93-2.29)	0.100			
ECOG PS≥2		2.01(1.09-3. 70)	0.025	1.95(1.06-3.62)		0.032
Smoking history		1.15 (0.89-1.49)	0.274			
Drinking history		1.12(0.87-1.44)	0.395			
Lung metastasis		0.91(0.61-1.36)	0.653			
Bone metastasis		1.62(0.94-2.80)	0.081			
Liver metastasis		1.12(0.80-1.58)	0.519			
Best response to first line						
PR		1				
SD		0.94(0.71-1.25)	0.689			
PD		0.98(0.69-1.39)	0.897			
Primary tumor location						
Cervical		1				
Upper		1.17(0.56-2.42)	0.677			
Middle		1.10(0.55-2.18)	0.793			
Lower		0.87(0.44-1.73)	0.701			
Radiotherapy		0.87(0.66-1.15)	0.333			
Treatment regimens of first-line						
ICIs monotherapy		1				
ICIs with Chemotherapy		1.14(0.56-2.31)	0.722			
ICIs with anti-VEGF		1.28(0.34-4.85)	0.717			
ICIs with both Chemotherapy and anti-VEGF		1.14(0.46-2.84)	0.782			
Discontinuation due to disease progression		1.89(1.19-2.98)	0.007	1.84(1.16-2.93)		0.010
Distant organ metastasis		1.15(0.88-1.50)	0.307			
Time to relapse after ICI-1 (months)						
<3		1				
≥3		0.88(0.68-1.14)	0.329			
Clinical T stage						
0-2		1				
3-4		1.16(0.80-1.70)	0.437			
Clinical N stage						
0-2		1				
3		1.86(1.40-2.45)	<0.001	1.73(1.31-2.30)		<0.001
						

PR: partial response; SD: steady disease; PD: progressive disease; ECOG PS: Eastern Cooperative Oncology Group Performance Status; HR: hazard ratio; CI: confidence interval.

**Table 4:** Short-term effect in total population.

Best response, n (%)	Rechallenge (n=211)	Non-rechallenge (n=118)	p-value
CR	0	0	
PR	50(23.7%)	11(9.3%)	
SD	107(50.7%)	54(45.8%)	
PD	54(25.6%)	53(44.9%)	
ORR	23.7%	9.3%	0.001
DCR	74.4%	55.1%	<0.001

CR: complete response; PR: partial response; SD: steady disease; PD: progressive disease; ORR: objective response rate; DCR: disease control rate.

**Table 5:** Adverse events

	No. (%) of patients			
	R(n=211)		NR(n=118)	
	Any grade	≥Grade 3	Any grade	≥Grade 3
Treatment-related adverse events^b^	136(64.5%)	37(17.5%)	78(66.1%)	22(18.6%)
Diarrhea	41(19.4%)	6(2.8%)	8(6.8%)	1(0.8%)
Vomiting	39(18.5%)	7(3.3%)	17(14.4%)	2(1.7%)
Fatigue	38(18.0%)	2(0.9%)	21(17.8%)	1(0.8%)
Neutropenia	36(17.1%)	4(1.9%)	20(16.9%)	4(3.4%)
Anorexia	27(12.8%)	3(1.4%)	16(13.6%)	2(1.7%)
Gastritis	26(12.3%)	3(1.4%)	8(6.8%)	1(0.8%)
Alopecia	24(11.4%)	0	15(12.7%)	0
Decreased white blood cells	23(10.9%)	3(1.4%)	13(11.0%)	6(5.1%)
Anemia	18(8.5%)	4(1.9%)	12(10.2%)	2(1.7%)
Decreased platelet count	18(8.5%)	2(0.9%)	12(10.2%)	3(2.5%)
Elevated liver enzymes	11(5.2%)	2(0.9%)	2(1.7%)	0
Mucositis/stomatitis	9(4.3%)	1(0.5%)	6(5.1%)	0
Fever	8(3.8%)	0	5(4.2%)	0
Arthritis/arthralgia/myalgia	8(3.8%)	0	3(2.5%)	0
Peripheral sensory neuropathy	6(2.8%)	0	0	0
Increased blood creatinine	5(2.4%)	0	2(1.7%)	0
Hyponatremia	5(2.4%)	0	0	0

a. Adverse events were classified according to Medical Dictionary for Regulatory Activities and graded according to the National Cancer Institute Common Terminology Criteria for Adverse Events, version 4.03. Grading ranges from 1 through 5 (1, mild; 2, moderate; 3, severe; 4. life-threatening; and 5, death). b. Treatment-related adverse events occurring in 1% or more of patients in either group are listed. Events are shown in descending order of frequency in R group. c. The numbers represent the number of patients with an adverse event.

## Abbreviations

AE: adverse events
CR: complete response
CI: confidence interval
DCR: disease control rate
ESCC: esophageal squamous cell cancer
ECOG PS: Eastern Cooperative Oncology Group Performance Status
HR: hazard ratio
ICI: immune checkpoint inhibitor
ORR: objective response rate
OS: overall survival
PD: progressive disease
PFS: progressive-free survival
PR: partial response
SD: steady disease
TRAE: treatment-related adverse event
