# Supplementary material for: Efficacy and Safety of Immune Checkpoint Inhibitor Rechallenge in the Treatment of Esophageal Squamous Cell Cancer
Source: J Cancer. 2025 Jan 1;16(3):943–51. doi: 10.7150/jca.104380 (PMC11705047; doi:10.7150/jca.104380)
Supplement: Supplementary file 1 — Supplementary table. [file jcav16p0943s1.pdf]

Supplementary Table 1. Treatment discontinuation caused by treatment-related adverse events.

	R(n=211)	NR(n=118)
Anemia	2(0.9%)	2(1.7%)
Diarrhea/colitis	2(0.9%)	0
Pneumonitis	1(0.5%)	0
Creatinine renal clearance decreased	1(0.5%)	1(0.8%)
Hypersensitivity	1(0.5%)	0
Neurotoxicity	2(0.9%)	0
Asthenia	2(0.9%)	1(0.8%)
Diarrhea	2(0.9%)	0
Hematemesis	1(0.5%)	0
Hypoesthesia	1(0.5%)	0
Platelet count decreased	0	1(0.8%)
Decreased appetite	0	1(0.8%)
Neutrophil count decreased	0	1(0.8%)
White blood cell count decreased	0	1(0.8%)
Total	15(7.1%)	8(6.8%)
